# Cetylpyridinium chloride and platinum nanoparticles effects in dogs with *Porphyromonas gulae*-infected periodontal disease

**DOI:** 10.1007/s11259-025-10945-z

**Published:** 2025-11-11

**Authors:** Tomoki Fukuyama, So Shirahata, Takahiro Miura, Kimika Odajima, Takashi Kobayashi, Risako Kawata, Masaru Murakami

**Affiliations:** 1 Laboratory of Veterinary Pharmacology, School of Veterinary Medicine, Azabu University, 1-17-71 Fuchinobe, Chuo-ku, Sagamihara-shi, Kanagawa 252-5201 Japan; 2 Center for Human and Animal Symbiosis Science, Azabu University, 1-17-71 Fuchinobe, Chuo-ku, Sagamihara-shi, Kanagawa 252-5201 Japan; 3 Primo Animal Hospital Kobuchi, 4-11-45, Higashi-Fuchinobe, Chuo-ku, Sagamihara-shi, Kanagawa Japan; 4 Primo Animal Hospital Sagamiohno, 2-4-8, Wakamatsu, Minami-ku, Sagamihara- shi, Kanagawa Japan

**Keywords:** Cetylpyridinium chloride, Platinum, Periodontal disease, *Porphyromonas gulae*, Dogs

## Abstract

Periodontal disease (PD) is a prevalent infectious condition in companion animals and represents an irreversible oral disorder; therefore, preventive dentistry is strongly recommended to inhibit and delay symptom progression. In this study, we investigated the synergistic effects of cetylpyridinium chloride (CPC) and platinum nanoparticles (PT) on the growth, halitosis, and inflammatory responses of the major canine PD pathogen *Porphyromonas gulae* (*P. gulae*) both in vitro and in a clinical study involving PD-affected dogs following dental scaling. The growth of *P. gulae* was significantly inhibited by CPC + PT treatment. Hydrogen sulfide and methyl mercaptan—key components of halitosis—produced by *P. gulae* were also significantly suppressed by the CPC and PT mixture, even after short-term treatment. Co-treatment with CPC and PT significantly inhibited *P. gulae*-induced pro-inflammatory cytokine production and SAPK/JNK phosphorylation in response to infection. These in vitro findings were corroborated by clinical results, in which daily oral administration of CPC + PT significantly suppressed the exacerbation of gingivitis, plaque adherence, halitosis, and PD pathogen activity following dental scaling. Our results suggest that daily CPC + PT treatment may be an effective approach to delay the progression of PD symptoms in affected dogs. There is no argument that mechanical dental care including regular brushing and dental scaling is necessary as the best way to prevent dental plaque formation. A dental rinse can be used as a suitable additional treatment to extend the interval between dental scaling procedures.

**Supplementary Information:**

The online version contains supplementary material available at 10.1007/s11259-025-10945-z.

## Introduction

Periodontal disease (PD) is one of the most prevalent infectious diseases in both humans and companion animals (Shirahata et al. 2023; Wallis et al. 2021). Disruption of the oral bacterial flora and an increase in highly pathogenic PD-associated bacteria are considered key factors in PD development. PD-associated bacterial infections are found in over 80% of adult dogs; however, the transmission routes of these highly pathogenic bacteria remain unclear, particularly in companion animals (Yasuda et al. 2024). PD is an irreversible oral condition, and damaged gingival tissue and alveolar bone typically do not regenerate (Yamasaki et al. 2012). Although dental implants and tissue engineering have been implemented in human dentistry, the use of artificial teeth is not practical in veterinary medicine due to the wide variety of anatomical structures in dogs and cats (De Rossi et al. 2025). Therefore, preventive dentistry—starting from an early life stage and including both hospital-based and home-based care—is strongly recommended to inhibit and delay symptom progression. However, general anesthesia is required for hospital-based procedures such as dental scaling in dogs and cats, making frequent veterinary care challenging, especially for elderly animals over 10 years of age, due to safety and economic concerns. As the companion animal population continues to age, there is increasing demand for at-home dental care products and methods (Ohira et al. 2024; Shirahata et al. 2024; Toyooka et al. 2025). In this study, we focused on cetylpyridinium chloride (CPC) and platinum nanoparticles (PT) to determine their synergistic effects on the growth, halitosis, and inflammatory responses of the major canine PD pathogen *Porphyromonas gulae* (*P. gulae*) in vitro. The preventive efficacy of the CPC + PT combination was also evaluated in a clinical study involving dogs with PD following dental scaling.

## Materials and methods

### Bactericidal effects of CPC and/or PT on P. gulae

CPC and PT used in this study were provided by QIX Corporation (Tokyo, Japan). The concentrations of CPC (0.00001%, 0.0001%) and PT (0.0012%, 0.012%) used in the in vitro experiments were based on the formulation of the dental rinse used in the clinical study (CPC: 0.0001%, PT: 0.012%). *P. gulae* strain ATCC 51,700 (fimA type A) was obtained from the Japan Collection of Microorganisms (RIKEN BioResource Research Center, Ibaraki, Japan) and cultured anaerobically on BD BBL™ CDC Anaerobic 5% Sheep Blood Agar (Becton, Dickinson and Company, NJ, USA). CPC and/or PT—Low: CPC 0.00001% + PT 0.0012%; High: CPC 0.0001% + PT 0.0012%—were co-incubated with *P. gulae* (1 ~ 5 × 10^8^ cfu/mL) for 5 min, 1 h, and 4 h. Bactericidal activity was evaluated using the BacTiter-Glo™ Microbial Cell Viability Assay (Promega KK, Tokyo, Japan) and measured with a luminometer (GloMax^®^-Multi Detection System, Promega KK). For bacterial inocula, overnight cultures of *P. gulae* were adjusted to an optical density at 600 nm (OD₆₀₀) corresponding to approximately 1 × 10⁸ CFU/mL, as determined by preliminary calibration curves. The stated ranges (e.g., 1–5 × 10⁸ CFU/mL for bactericidal assays and 1–5 × 10⁶ CFU/mL for infection assays) reflect minor variability inherent to anaerobic bacterial culture and enumeration. For all experiments, inocula were prepared in the same manner, and equivalent OD₆₀₀ values were used to ensure comparability across replicates. All experiments included three independent biological replicates (different passages of bacteria or cells), with each biological replicate containing 3–6 technical replicates. Data are presented as representative results or as mean ± SEM of all replicates. Data are presented as representative results or as mean ± SEM of the technical replicates. We confirmed that all experiments showed a similar tendency, and representative results are shown in the figures.

### Inhibitory effects of CPC and/or PT on hydrogen sulfide and methyl mercaptan production by P. gulae

Cultured *P. gulae* (1 ~ 5 × 10^8^ cfu/mL) was co-incubated with CPC and/or PT for 5 min. The production of hydrogen sulfide and methyl mercaptan was measured using a gas chromatography system (OralChroma, Nissha FIS, Inc., Tokyo, Japan). Each condition was evaluated using four independent biological replicates, each with four technical replicates, to ensure reproducibility. Data are presented as representative results or as mean ± SEM of the technical replicates. We confirmed that all experiments showed a similar tendency, and representative results are shown in the figures.

### Inhibitory effects of CPC and/or PT on pro-inflammatory cytokine secretion and phosphorylation of SAPK/JNK by P. gulae-induced canine macrophage cell line

The DH82 canine macrophage cell line was obtained from the American Type Culture Collection (Manassas, VA, USA) and cultured in Eagle’s Minimum Essential Medium (EMEM; FUJIFILM Wako Pure Chemical Corporation, Osaka, Japan) supplemented with 10% fetal calf serum (FCS; Sigma-Aldrich Co. LLC, Tokyo, Japan) and penicillin–streptomycin (FUJIFILM Wako Pure Chemical Corporation). Cells (1 × 10^4^ cells/100 µL) were exposed to CPC and/or PT and *P. gulae* (1 ~ 5 × 10^6^ cfu/mL) for 24 h (cytokine assay) or 1 h (Western blot analysis). The levels of IL-1β, IL-6, and TNF-α in the supernatant of DH82 cells were assessed using an enzyme-linked immunosorbent assay (ELISA; DuoSet ELISA kit, R&D Systems, Minneapolis, MN, USA). Optical density [OD] was measured using a microplate reader. The phosphorylation levels of p46 and p54 in DH82 cells were evaluated by Western blot analysis. Total proteins (30 µg), extracted using the M-PER™ Mammalian Protein Extraction Reagent (Thermo Fisher Scientific, Inc., Kanagawa, Japan), were separated by SDS-PAGE and transferred to PVDF membranes using the Trans-Blot Turbo Transfer System (Bio-Rad Laboratories, Inc., Tokyo, Japan). Primary antibodies (anti-phospho-SAPK/JNK and anti-SAPK/JNK; Cell Signaling Technology, Inc., Danvers, MA, USA) were used for detection. Bands were visualized using a secondary antibody and detected with ImmunoStar^®^ Zeta (FUJIFILM Wako Pure Chemical Corporation). Protein bands were quantified using the iBright Imaging System (Thermo Fisher Scientific). For each assay, three independent biological replicates were performed, each including 7 (Cytokine assay) or 4 (Western blot analysis) technical replicates. Data are presented as representative results or as mean ± SEM of the technical replicates. We confirmed that all experiments showed a similar tendency, and representative results are shown in the figures.

## Daily oral CPC and PT treatments in dogs with P. gulae-positive PD after dental scaling

All experimental protocols were approved by the Animal Care and Use Program of Azabu University (Approval No. 200318-1). The study design is illustrated in Fig. 3A. The study included 34 dogs diagnosed with moderate to severe PD (information of the dogs were shown in Table S1). All dogs underwent dental scaling under general anesthesia at Primo Animal Hospital Kobuchi (Kanagawa, Japan) or Primo Animal Hospital Sagamiohno (Kanagawa, Japan) and were subsequently assigned to groups based on owner consent and treatment compliance feasibility, which resulted in uneven allocation: an untreated group (11 dogs) or a treatment group receiving daily dental rinse (Q-NESS MOUTH CLEANER, QIX Corporation, Tokyo, Japan). Although no formal power analysis was performed prior to enrollment, the sample size was comparable to similar exploratory veterinary clinical studies and sufficient to detect statistically significant differences in key clinical outcomes. The dental rinse contained 0.0001% CPC and 0.012% PT and was administered once daily for 30 min after the evening meal for three months. All dogs in the dental rinse group accepted the dental rinse at least 30 mL every day in drinking water (without mechanical swabbing or rinsing). Prior to the clinical trial, safety assessment of dental rinse was performed in mice *via* acute oral administration study and 28 days oral administration study (data not shown). Oral lethal dose 50 in mice was higher than 2000 mg/kg and there was no general toxicity when dental rinse was orally administered for 28 days. There were no dental rinse related clinical issues during the experimental period. All dogs were not offered any meals until the next morning after the dental rinse. Plaque accumulation and gingivitis were visually evaluated based on the most severely affected tooth using a 0–3 scale (0 = clinically normal, 1 = gingivitis only, without attachment loss, 2 = early periodontitis, 3 = advanced periodontitis) without anesthesia or radiographs (Ohira et al. 2024; Toyooka et al. 2025). Halitosis was subjectively evaluated using the same 0–3 scale. Enzymatic activity for hydrolyzing N-benzoyl-DL-arginine-naphthylamide (BANA)—a marker for canine periodontal pathogens such as *Porphyromonas gingivalis* and *P. gulae*—was assessed using the AD*plit* kit (Kyoritsu Seiyaku Corporation, Tokyo, Japan) (Ardila et al. 2023). All treatments and evaluations were performed in a double-blinded manner. Clinical evaluations were conducted at baseline (prior to scaling), and at 1, 4, and 12 weeks after scaling. The week-1 assessment was included to capture the short-term effect of dental scaling. The 4- and 12-week time points were chosen as clinically relevant intervals based on previous reports that plaque and gingivitis typically recur within 2–6 weeks after scaling. These time points therefore allowed us to assess both the early recurrence and the longer-term preventive effects of CPC + PT treatment.

### Statistical analyses

All data are expressed as the mean ± standard error of the mean (SEM). For the in vitro experiments, multigroup comparisons were analyzed using analysis of variance (ANOVA) followed by Dunnett’s multiple comparison test. For the clinical study, two-way ANOVA was used, followed by the uncorrected Fisher’s least significant difference (LSD) test. Statistical significance was set at *p* < 0.05. All analyses were conducted using GraphPad Prism 10 (GraphPad Software, San Diego, CA, USA).

## Results

**Fig. 1:**
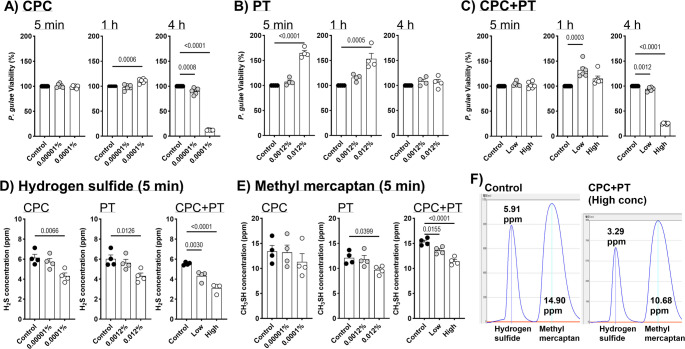
Direct effects of CPC and/or PT treatment on *P. gulae* activity. Bactericidal effects of (**a**) CPC alone, (**b**) PT alone, and (**c**) CPC + PT combination against *P. gulae* at each time point. (**d**) Hydrogen sulfide and (**e**) methyl mercaptan production by *P. gulae* following 5-min co-incubation with CPC and/or PT. (f) Representative gas chromatography images. Each result represents the mean (% or ppm) ± 1 SEM. *n* = 4–6 per group. *p* < 0.05 (Dunnett’s multiple comparison test) vs. control (0%) group. CPC, cetylpyridinium chloride; *P. gulae*, *Porphyromonas gulae*; PT, platinum nanoparticles

**Fig. 2:**
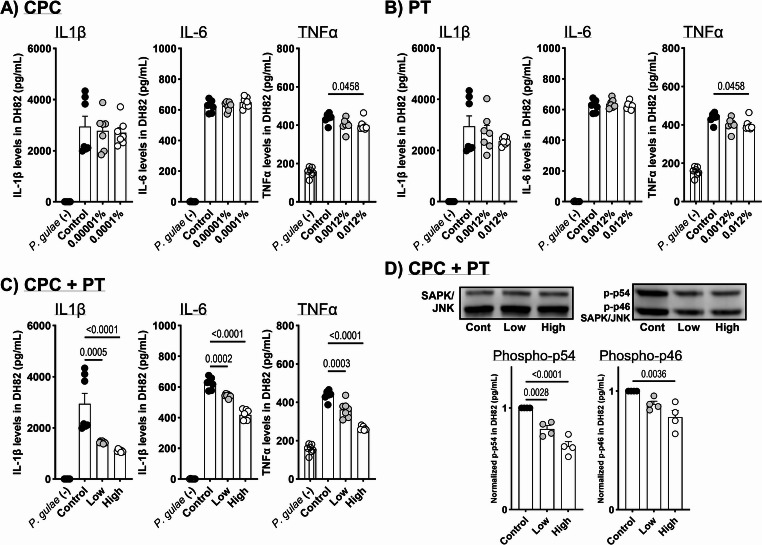
IL-1β, IL-6, and TNF-α secretion by *P. gulae*-infected DH82 cells after treatment with (**a**) CPC alone, (**b**) PT alone, and (**c**) CPC + PT combination. (**d**) Representative Western blot images. Each result represents the mean (pg/mL) ± 1 SEM. *n* = 4 (Western blot) or 7 (cytokine assay) per group. *p* < 0.05 (Dunnett’s multiple comparison test) vs. *P. gulae*-infected control group. IL, interleukin; TNF, tumor necrosis factor

The proliferative activity of the canine periodontal pathogen *P. gulae* was significantly inhibited at 4 h post-treatment with 0.0001% CPC (Fig. 1A); however, the growth of *P. gulae* was significantly increased at 5 min and 1 h post-treatment with 0.0001% CPC (Fig. 1B). In the CPC + PT treatment groups, a significant increase in the growth of *P. gulae* was observed at 1 h post-treatment with the low concentration, whereas significant decreases in growth were observed at 4 h post-treatment with both low and high concentrations (Fig. 1C). In this study, hydrogen sulfide and methyl mercaptan—major components of halitosis produced by *P. gulae*—were measured by gas chromatography. Hydrogen sulfide production was significantly suppressed by high concentrations of CPC and/or PT, and by a mixture of low concentrations of CPC and PT, even after short-term treatment (5 min) (Fig. 1D and F). Methyl mercaptan production was not affected by CPC alone. However, high concentrations of PT resulted in significant suppression compared to the control group. Notably, the combination of CPC and PT synergistically decreased methyl mercaptan production in a dose-dependent manner (Fig. 1E and F). To investigate the inhibitory effects of CPC and/or PT on inflammatory responses in canine macrophages (DH82), production of representative pro-inflammatory cytokines (IL-1β, IL-6, and TNF-α) and phosphorylation of SAPK/JNK induced by *P. gulae* infection were assessed. A statistically significant but minimal reduction in TNF-α production was observed with high concentrations of CPC or PT after 24-h exposure to *P. gulae* (Fig. 2A and B). In contrast, co-treatment with CPC and PT significantly inhibited *P. gulae*-induced IL-1β, IL-6, and TNF-α production in a dose-dependent manner (Fig. 2C). To further confirm the inhibitory effects of the CPC + PT combination on cytokine production, the involvement of MAPKs, including SAPK/JNK, was assessed via phosphorylation analysis. The upregulated phosphorylation of SAPK/JNK in response to *P. gulae* was significantly inhibited by co-treatment with CPC and PT in a dose-dependent manner (Fig. 2D).

**Fig. 3:**
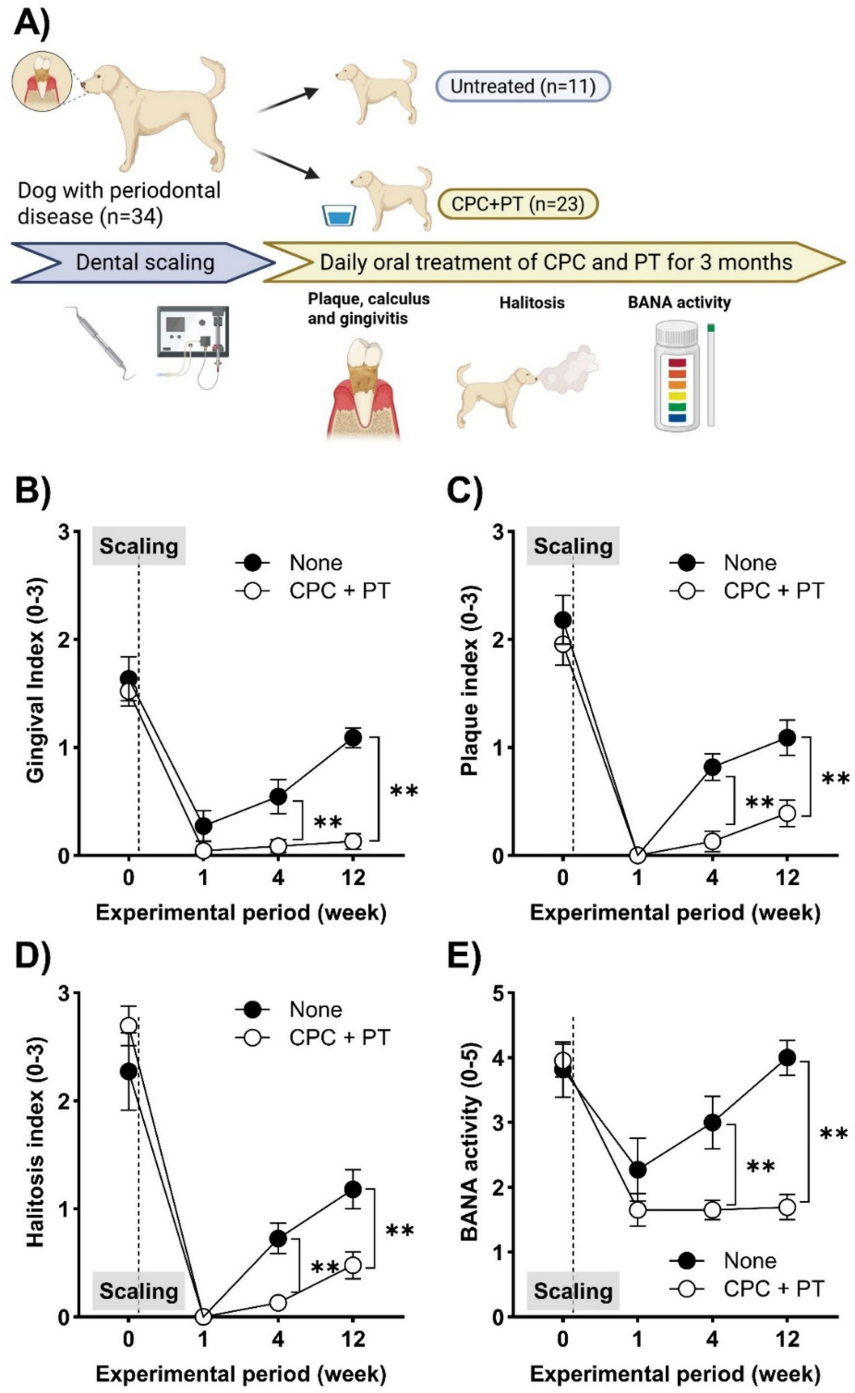
(**a**) Experimental schedule and study groups in clinical study. (**b**) Gingivitis, (**c**) plaque adherence, (**d**) halitosis, and (**e**) periodontal pathogen activity were significantly ameliorated by CPC + PT treatment compared to the control group. Each result represents the mean ± 1 SEM. **p < 0.01 (uncorrected Fisher’s least significant difference test). BANA, N-benzoyl-DL-arginine-naphthylamide

To demonstrate the effects of the CPC + PT combination on the progression of PD, dogs with PD who had undergone dental scaling were treated with a dental rinse containing CPC and PT for three months. Chronic treatment with CPC and PT significantly inhibited the exacerbation of gingivitis, plaque adhesion, halitosis, and the activity of periodontal pathogens, including *P. gulae*, compared to the untreated group (Fig. 3B–E, Table S3). As shown in Table S2, plaque and gingivitis indices decreased markedly in both groups at week 1 after scaling. This reduction reflects the immediate short-term effect of full-mouth dental scaling, which temporarily reduces plaque accumulation and gingival inflammation regardless of subsequent treatment. Group differences became apparent from week 4 onward, when recurrence of PD symptoms began to diverge between treated and untreated dogs.

## Discussion

Our findings indicate that the combination of CPC and PT synergistically suppressed *P. gulae* growth, as well as hydrogen sulfide and methyl mercaptan production—major components of halitosis—generated by *P. gulae*. In addition, direct exposure to the CPC + PT combination inhibited inflammatory cytokine production and MAPK signaling phosphorylation in canine macrophages following *P. gulae* infection. Furthermore, chronic treatment with a CPC- and PT-containing dental rinse significantly prevented the exacerbation of PD symptoms, including gingivitis, plaque adherence, halitosis, and periodontal pathogen activity.

CPC is a surfactant known for reducing bacterial surface tension, and previous in vitro studies have demonstrated its bactericidal activity against PD pathogens (Figuero et al. 2023). Moreover, the use of CPC as an oral rinse has been reported to prevent plaque formation, maintain oral hygiene, and reduce oral malodor (Belibasakis et al. 2023). PT is also recognized for its bactericidal and deodorizing properties (Itohiya et al. 2019), with confirmed in vitro activity against bacteria and organic matter (Itohiya et al. 2019). Wu et al. (2021) further demonstrated that PT possesses strong biofilm elimination capabilities against *Staphylococcus aureus* and *Escherichia coli*. However, these findings have primarily focused on human pathogens, and the efficacy of CPC and PT in canine PD had not been previously investigated. In addition, gargle-type mouthwashes and dental gels are not suitable for use in dogs and cats. Therefore, the development of veterinary dental care products requires not only evidence of efficacy but also a strong emphasis on safety, including concentration and administration optimization. In this study, we evaluated and optimized the effectiveness of CPC and PT in alleviating canine PD-associated symptoms.

The concentrations of CPC and PT used in this study were based on those present in the final product and selected to ensure the absence of adverse effects during clinical use. The safety of these concentrations was also confirmed in our clinical study. When comparing the bactericidal effects of CPC and/or PT against the representative canine periodontal pathogen *P. gulae*, CPC alone and the CPC + PT combination significantly suppressed bacterial growth, whereas PT alone had no observable effect. Although direct antibacterial effects of PT have been demonstrated in previous studies (Itohiya et al. 2019), its efficacy may vary substantially depending on concentration and bacterial species. *P. gulae* is isolated from the oral microbiome of dogs with PD, and several epidemiological studies have shown that *P. gulae* infection correlates with PD severity and the number of permanent teeth in dogs (Shirahata et al. 2023; Yasuda et al. 2024). While PT alone had no effect on *P. gulae* growth, hydrogen sulfide and methyl mercaptan production by *P. gulae* was significantly suppressed by both PT alone and the CPC + PT combination. These results suggest a clear synergistic effect of the CPC + PT combination. Similar synergistic effects were observed in *P. gulae*-induced cytokine secretion in the canine macrophage cell line. Neither CPC nor PT alone significantly reduced IL-1β or IL-6 production; however, the CPC + PT combination significantly inhibited the production of these inflammatory cytokines in a dose-dependent manner. The observed inhibition of MAPK phosphorylation by the CPC + PT combination further supports the inhibitory pattern of cytokine production.

Finally, a clinical study in dogs with PD was conducted to evaluate the efficacy of CPC + PT treatment in preventing the progression of periodontal symptoms following dental scaling. Daily oral administration of CPC + PT significantly inhibited the exacerbation of gingivitis, plaque adherence, halitosis, and periodontal pathogen activity. The early decline in indices at week 1 reflected the immediate outcome of scaling, while the re-emergence of group differences by weeks 4 and 12 highlights the preventive effect of CPC + PT in delaying recurrence. The choice of these intervals aligns with clinical observations that plaque and gingivitis generally reappear within 2–6 weeks after scaling in dogs, making them appropriate benchmarks for evaluating treatment efficacy. These results offer a significant advantage for both veterinarians and pet owners, as CPC + PT treatment may help extend the interval between dental scaling procedures requiring general anesthesia. The interval between the dental scaling procedures should be variable depending on the stage of PD and anatomical situation of dogs, but 6 months (24 weeks) will be needed until reaching index 2 of dental plaque (the timing to consider the next dental scaling) after the dental scaling according to our results. Using the dental rinse fortunately extends for another 6 months until reaching index 2 of dental plaque. On the other hand, solo treatment of current dental rinse did not remove the dental plaque and ameliorate the PD conditions in dogs with severe PD (data not shown). This limitation may be related to the type of dental care product used (e.g., dental gel, spray, or supplement); nevertheless, some form of mechanical dental care appears to be necessary for optimal effect. The owners often neglect regular brushing of their dogs´ teeth; however, there is no argument that mechanical dental care including regular brushing and dental scaling is necessary, because it is the best way to prevent dental plaque formation. A dental rinse can be used as a suitable additional treatment. This study also has other limitations, including the relatively small sample size, uneven group allocation, and the absence of an a priori power analysis. In addition, body weight and obesity status, known risk factors for PD, were not monitored in this trial. Future studies with larger, balanced cohorts and inclusion of obesity-related parameters will be required to validate and expand upon these findings. Despite this, our findings strongly suggest that daily treatment with the CPC + PT combination may be a useful approach for preventing the exacerbation of PD symptoms in young dogs or in those recovering from dental scaling.

## Supplementary Information

Below is the link to the electronic supplementary material.

Supplementary Material 1 (PDF 194 KB)

Supplementary Material 2 (PNG 81.5 KB)

High Resolution Image (TIF 9.78 MB)

Supplementary Material 3 (DOCX 59.2 KB)

## Data Availability

The data are available from the corresponding author upon reasonable request.
