# Supplementary figures and images for: Cetylpyridinium chloride and platinum nanoparticles effects in dogs with Porphyromonas gulae-infected periodontal disease
Source: Vet Res Commun. 2025 Nov 11;50(1):26. doi: 10.1007/s11259-025-10945-z (PMC12605614; doi:10.1007/s11259-025-10945-z)

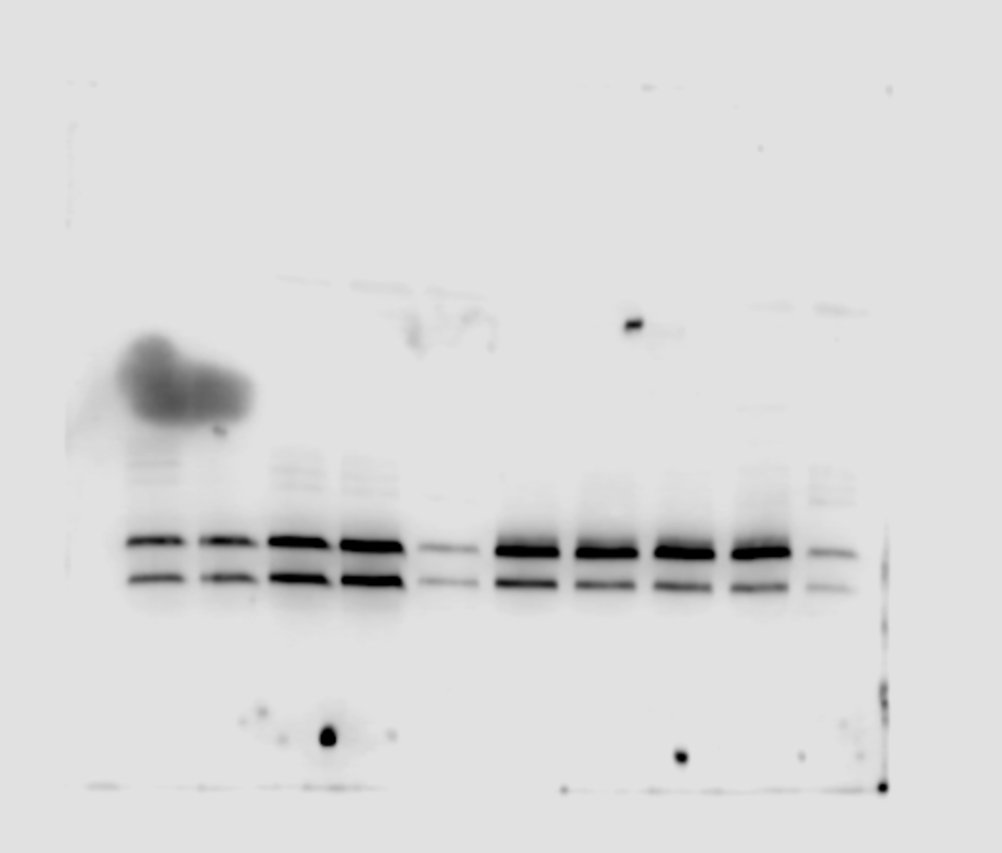

Supplement: Supplementary file 2 — Supplementary Material 2 (PNG 81.5 KB) [file 11259_2025_10945_Fig4_ESM.png]

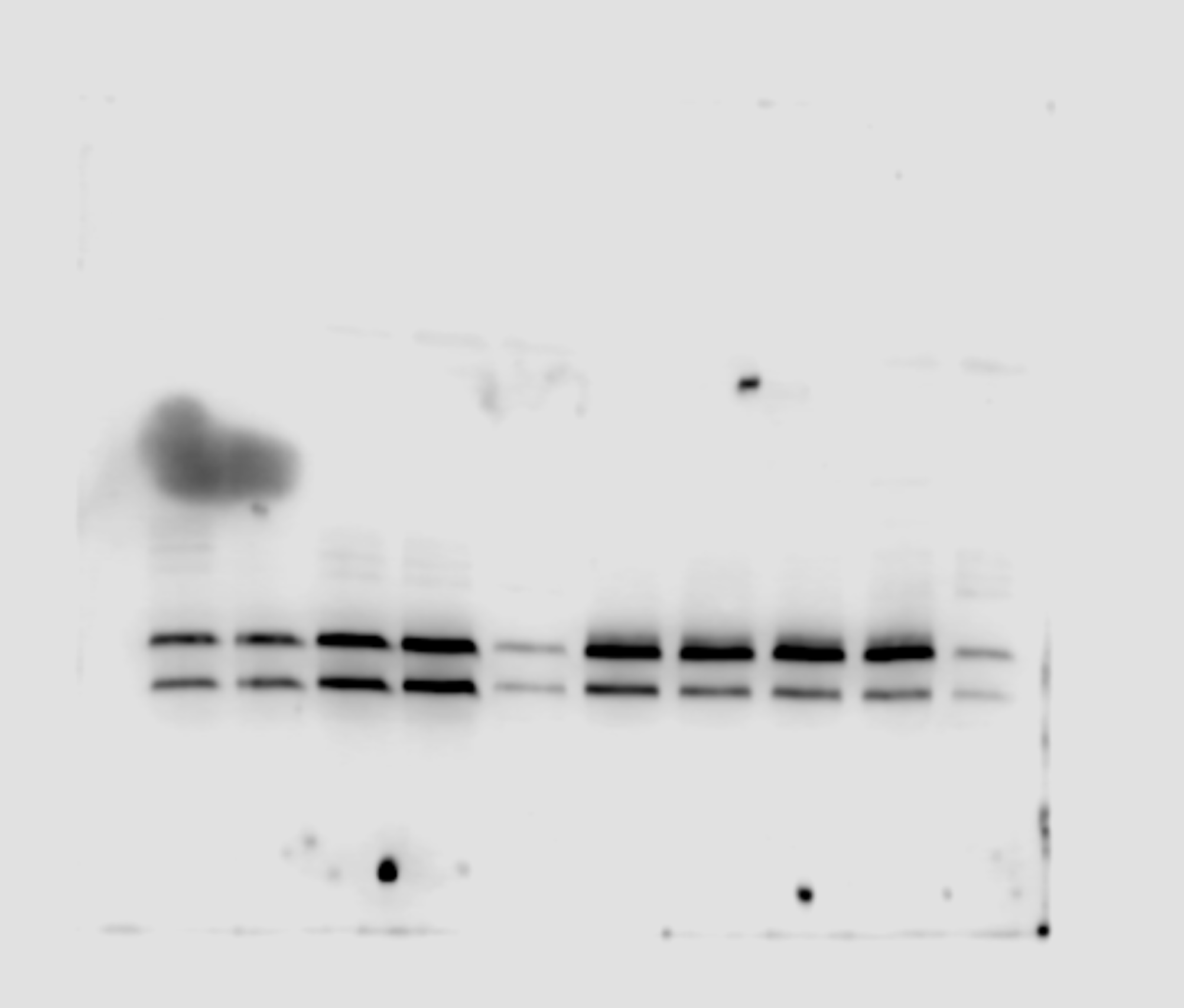

Supplement: Supplementary file 3 — High Resolution Image (TIF 9.78 MB) [file 11259_2025_10945_MOESM2_ESM.tif]
