# Supplementary material for: Cetylpyridinium chloride and platinum nanoparticles effects in dogs with Porphyromonas gulae-infected periodontal disease
Source: Vet Res Commun. 2025 Nov 11;50(1):26. doi: 10.1007/s11259-025-10945-z (PMC12605614; doi:10.1007/s11259-025-10945-z)
Supplement: Supplementary file 4 — Supplementary Material 3 (DOCX 59.2 KB) [file 11259_2025_10945_MOESM3_ESM.docx]

TABLE S1 Dog breeds included in the clinical study

| Group | Breeds | Age | Sex | Anamnesis (in the past year) |
| --- | --- | --- | --- | --- |
| Untreated group  (n=11) | Toy Poodle | 9 years | Male (castration) | None |
|  | Shiba | 7 years | Female (spay) | None |
|  | Toy Poodle | 6 years | Male (castration) | Food allergy (special diet) |
|  | Toy Poodle | 10 years | Female (spay) | None |
|  | Miniature Dachshund | unknown | Female (spay) | Eczema |
|  | Chihuahua | 5 years | Female (spay) | None |
|  | Miniature Dachshund | 7 years | Female (spay) | None |
|  | Chihuahua | 9 years | Male (castration) | Patella |
|  | Chihuahua | 8 years | Female (spay) | None |
|  | Beagle | 10 years | Female (spay) | Food allergy (special diet) |
|  | Italian Greyhound | 6 years | Male (castration) | None |
| CPC+PT group  (n=23) | Yorkshire terrier | 10 years | Female (spay) | None |
|  | Toy Poodle | 5 years | Male (castration) | None |
|  | Maltese | 5 years | Male (castration) | None |
|  | Miniature Dachshund | unknown | Female (spay) | Otitis |
|  | Miniature Dachshund | 5 years | Male (castration) | None |
|  | Toy Poodle | 8 years | Male (castration) | None |
|  | Miniature Dachshund | 7 years | Female (spay) | None |
|  | Chihuahua | 6 years | Male (castration) | Food allergy (special diet) |
|  | Pomeranian | 5 years | Female (spay) | None |
|  | Italian Greyhound | 9 years | Female (spay) | Arthritis |
|  | Pomeranian | 11 years | Male (castration) | None |
|  | Yorkshire terrier | 8 years | Female (spay) | None |
|  | Miniature Dachshund | 9 years | Female (spay) | None |
|  | Miniature Dachshund | 8 years | Female (spay) | None |
|  | Toy Poodle | 5 years | Male (castration) | None |
|  | Toy Poodle | 6 years | Male (castration) | None |
|  | Chihuahua | 11 years | Female (spay) | Otitis |
|  | Chihuahua | 8 years | Female (spay) | None |
|  | Toy Poodle | 4 years | Female (spay) | None |
|  | Jack Russell | 7 years | Male (castration) | Eczema |
|  | Italian Greyhound | 8 years | Female (spay) | None |
|  | Pomeranian | 7 years | Male (castration) | None |
|  | Jack Russell | 8 years | Male (castration) | None |

TABLE S2 Plaques and gingivitis indexes in each dog

| Group | Plaque index | | | | Gingivitis index | | | |
| --- | --- | --- | --- | --- | --- | --- | --- | --- |
|  | 0 wk | 1 wk | 4 wk | 12 wk | 0 wk | 1 wk | 4 wk | 12 wk |
| Untreated group  (n=11) | 2 | 0 | 0 | 1 | 1 | 0 | 0 | 1 |
|  | 3 | 0 | 1 | 2 | 3 | 0 | 1 | 2 |
|  | 2 | 0 | 1 | 1 | 2 | 1 | 0 | 1 |
|  | 2 | 0 | 1 | 2 | 2 | 1 | 1 | 1 |
|  | 2 | 0 | 1 | 1 | 2 | 0 | 1 | 1 |
|  | 3 | 0 | 1 | 1 | 2 | 1 | 1 | 1 |
|  | 2 | 0 | 1 | 1 | 1 | 0 | 1 | 1 |
|  | 1 | 0 | 0 | 0 | 1 | 0 | 0 | 1 |
|  | 3 | 0 | 1 | 1 | 1 | 0 | 1 | 1 |
|  | 3 | 0 | 1 | 1 | 2 | 0 | 0 | 1 |
|  | 1 | 0 | 1 | 1 | 1 | 0 | 0 | 1 |
| CPC+PT group  (n=23) | 3 | 0 | 0 | 1 | 2 | 0 | 0 | 0 |
|  | 2 | 0 | 0 | 1 | 2 | 0 | 0 | 0 |
|  | 0 | 0 | 0 | 1 | 2 | 0 | 1 | 1 |
|  | 1 | 0 | 0 | 0 | 2 | 0 | 0 | 0 |
|  | 2 | 0 | 0 | 0 | 2 | 0 | 0 | 0 |
|  | 3 | 0 | 0 | 0 | 2 | 0 | 0 | 0 |
|  | 3 | 0 | 0 | 0 | 1 | 0 | 1 | 0 |
|  | 1 | 0 | 0 | 1 | 2 | 0 | 0 | 0 |
|  | 1 | 0 | 1 | 1 | 2 | 0 | 0 | 0 |
|  | 2 | 0 | 0 | 0 | 2 | 0 | 0 | 0 |
|  | 3 | 0 | 0 | 0 | 0 | 0 | 0 | 0 |
|  | 1 | 0 | 0 | 0 | 1 | 0 | 0 | 0 |
|  | 1 | 0 | 0 | 0 | 2 | 1 | 0 | 1 |
|  | 2 | 0 | 0 | 0 | 2 | 0 | 0 | 0 |
|  | 1 | 0 | 0 | 0 | 3 | 0 | 0 | 1 |
|  | 3 | 0 | 0 | 1 | 1 | 0 | 0 | 0 |
|  | 1 | 0 | 0 | 1 | 1 | 0 | 0 | 0 |
|  | 3 | 0 | 0 | 0 | 1 | 0 | 0 | 0 |
|  | 3 | 0 | 0 | 0 | 1 | 0 | 0 | 0 |
|  | 2 | 0 | 0 | 0 | 1 | 0 | 0 | 0 |
|  | 3 | 0 | 0 | 0 | 1 | 0 | 0 | 0 |
|  | 2 | 0 | 2 | 2 | 1 | 0 | 0 | 0 |
|  | 2 | 0 | 0 | 0 | 1 | 0 | 0 | 0 |
